# Conserving Critical Sites for Biodiversity Provides Disproportionate Benefits to People

**DOI:** 10.1371/journal.pone.0036971

**Published:** 2012-05-30

**Authors:** Frank W. Larsen, Will R. Turner, Thomas M. Brooks

**Affiliations:** 1 Science & Knowledge Division, Conservation International, Arlington, Virginia, United States of America; 2 Center for Macroecology, Evolution and Climate, University of Copenhagen, Copenhagen, Denmark; 3 NatureServe, Arlington, Virginia, United States of America; 4 School of Geography and Environmental Studies, University of Tasmania, Hobart, Australia; 5 World Agroforestry Center, University of the Philippines Los Baños, Laguna, Philippines; University of Otago, New Zealand

## Abstract

Protecting natural habitats in priority areas is essential to halt the loss of biodiversity. Yet whether these benefits for biodiversity also yield benefits for human well-being remains controversial. Here we assess the potential human well-being benefits of safeguarding a global network of sites identified as top priorities for the conservation of threatened species. Conserving these sites would yield benefits – in terms of a) climate change mitigation through avoidance of CO_2_ emissions from deforestation; b) freshwater services to downstream human populations; c) retention of option value; and d) benefits to maintenance of human cultural diversity – significantly exceeding those anticipated from randomly selected sites within the same countries and ecoregions. Results suggest that safeguarding sites important for biodiversity conservation provides substantial benefits to human well-being.

## Introduction

Conserving important sites for biodiversity is essential to meet internationally agreed goals of preventing species extinctions and slowing biodiversity loss [1]. Conservation also holds the potential to benefit people [2] either through direct provision of ecosystem services, or through financial compensation to local communities for safeguarding ecosystem services. Yet beyond their value for safeguarding species *per se*, however, controversy exists on whether safeguarding sites that benefit biodiversity also delivers benefits to human communities [3], [4]. Assessing the net benefits of conservation requires that we quantify the ecosystem service benefits that would be delivered by conserving these sites [2], [5], as well as the costs of conserving them. Previous analyses have explored the concordance between biodiversity conservation priority and ecosystem service value, at global scales [6]–[9], national/regional scales [10]–[12], and for single sites [13], with mixed findings.

However, this question has never been addressed for a global network of priority sites for biodiversity conservation identified using consistent criteria and widespread enough to illuminate general patterns. Here, as an example of a comprehensive global network of priority sites, we focused on those identified by the Alliance for Zero Extinction [14] as holding the last remaining population of one or more Endangered or Critically Endangered species [15]. This network of critical sites for global species conservation serves as an important blueprint for targeted conservation action for species, for example in informing actions by the Global Environmental Facility (GEF) and the World Bank (http://www.thegef.org/gef/press_release/CBD_COP10_AZE). Thus, this investigation of the joint biodiversity and ecosystem service roles of these sites is a key step in guiding the planning of these and other funding bodies. Here we assessed the ecosystem service benefits delivered by protection of this global network of priority sites. We assessed whether protection of such a global network of sites (n = 524) delivers disproportionate ecosystem service benefits compared to appropriately constructed null models. We compared the aggregate delivery of ecosystem services predicted to ensue from safeguarding all priority sites to that expected from conservation of other sites within those countries (n = 96 countries) containing priority sites. Although countries are particularly relevant units for such comparisons, the large size and heterogeneity of some countries could distort results, and so we also compared results for priority sites to those expected at random from conservation within those terrestrial ecoregions (n = 325 ecoregions) containing priority sites. We focus this study on the aggregated delivery of ecosystem services because of the uncertainty for values of specific sites due to the use of global datasets.

The question of whether conserving sites of importance for biodiversity conservation is of much more than academic interest. For example, Aichi Target 11 of the Convention on Biological Diversity demands that “by 2020, at least 17 per cent of terrestrial and inland water, and 10 per cent of coastal and marine areas, especially areas of particular importance for biodiversity and ecosystem services, are conserved…” [16]. Our null models test the benefits of implementing just the high level component of this target – reaching a percentage (17% in the case of land areas) coverage of protected areas without reference to where these are located (that is, at random). By contrast, our analyses of the potential ecosystem service benefits delivered by safeguarding Alliance for Zero Extinction sites inform the implementation of Aichi Target 11 in full.

We focused on four types of benefits to human well-being – carbon storage, provision of freshwater ecosystem services, option value, and cultural value – for which global data are available. These four classes of benefits span a range of ecosystem service types as classified by the Millennium Ecosystem Assessment [2], including regulating (carbon), provisioning (freshwater), and cultural services as well as option value. They also span a range in the spatial scales over which people benefit: global (carbon, option value), regional (freshwater), and local (cultural).

Deforestation, particularly in the tropics, is a major contributor to global CO_2_ emissions [17] and protected areas may be effective means to reduce these emissions [18]. Because curbing deforestation is suggested to be a comparatively inexpensive means of reducing CO_2_ emissions [19], a global financial mechanism to reduce emissions from deforestation and forest degradation in developing countries (Reducing Emissions from Deforestation and forest Degradation, or REDD+) has been established under the ‘Cancun Agreements’ of the United Nations Framework Convention on Climate Change [20]. Thus, funding in a REDD+ mechanism could be well beyond existing conservation funding with substantial consequent potential to influence global forest conservation and benefit local people through REDD+ payments.

Many terrestrial ecosystems are important for the freshwater services they provide to people downstream, and in particular for ensuring the delivery of clean water [8], [21]. We therefore use two measures of freshwater services to downstream human populations: water quality and potential water provision.

Option value is the as-yet-unknown benefit that conservation of biodiversity provides for current and future generations (e.g., drug discovery). For example, the extinction of gastric-brooding frogs of the genus *Rheobatrachus* resulted in lost options for potential treatment of ulcers that affect millions of humans worldwide [22]. Evolutionary distinctiveness is one measure of the future options, or ‘option value’, that conservation of the biodiversity at a site represents [23].

In the broader sense the concept of cultural services would encompass ecosystems that contribute to the nonmaterial benefits that arise from human-ecosystem relationships, including recreational experiences, sense of place, and others [24]. The cultural value of biodiversity is difficult to measure – especially on a global scale – and therefore efforts to analyze cultural diversity have focused on language diversity [25]. Language richness clearly does not represent all cultural diversity, which in turn may not directly correspond to the cultural values of biodiversity *per se* [25]. Nevertheless, language richness quantitatively indicates one important aspect of the relationship between human cultures and nature [25]–[27]. Local and indigenous languages are also the repositories of traditional knowledge about the environment and its systems [2]. Thus, we used human language [28] as a proxy for the cultural value that conserving nature provides [29], given that almost two-thirds of the world's languages belong to forest-dwelling people and that the cultural identity and value systems of many indigenous and traditional people are shaped by close interaction with the natural environment [2].

An important next step is to estimate the financial value of these ecosystem services for comparison with conservation costs [30]. While it is still not possible to undertake solid economic analyses at this scale, we can provide some coarse estimates to illustrate the magnitude of the economic benefits versus costs of protecting this global network of priority sites. We therefore estimated the potential financial yield from avoided carbon emissions from deforestation at priority sites in comparison to the estimated cost of protecting these sites. We estimated the cost of safeguarding the network of priority sites in developing countries as protected areas by using site and country characteristics to predict ongoing management costs [31]; and using agricultural rents to predict acquisition costs [32].

## Methods

### Boundaries for priority sites

As only limited data exist for the full set of 595 Alliance for Zero Extinction sites [14] we derived boundaries around the site locality points based on existing polygon layers in the following hierarchical way:

- Alliance for Zero Extinction sites [14] (boundaries previously delineated for 102 sites),
- Key Biodiversity Areas [33], [34], where these have been identified (58 additional sites),
- Important Bird Areas [35], where these have been identified (127 additional sites),
- Protected areas in the World Database of Protected Areas [36] (national sites have precedence over international sites) (101 additional sites),
- Species ranges (extent of occurrence) for the Alliance for Zero Extinction trigger species for amphibians [15], [37], mammals [15], [38] and threatened birds [35] (85 additional sites). If ranges of several trigger species were available for a priority site location we used the merged ranges.

We dealt with potential inaccuracies in georeferencing of original locality points by making sure the selected polygons were supported by trigger species ranges and Alliance for Zero Extinction tabular information on either protected area or Important Bird Area name (when available). Boundaries for the remaining points (51 sites) were derived by using circle polygons of median priority area size (26,963 ha). This procedure resulted in 524 site polygons with mean area of 113,079±277,550 ha (s.d.). This differs from the original 595 sites [14] because some polygons contain more than one priority site.

### The four ecosystem services

#### Carbon storage

Carbon stock was estimated by using a global map of biomass carbon stored in above- and belowground living vegetation with a resolution of 0.0089 decimal degrees [39] and restricted to carbon in natural land covers by using a global land cover layer [40]. Despite limitations in the global map of biomass carbon [39], this is the only globally consistent dataset on vegetation biomass carbon. We also estimated potential annual CO_2_ emissions avoided from deforestation as follows: carbon density (t C/ha)×area (ha)×estimated deforestation rate (%/yr)×3.66 CO_2_ equivalents. We used national deforestation rates [41] as no spatially explicit deforestation data are globally available at sufficient resolution. We used national deforestation rates for countries above global mean (0.22%/yr). However, for sites in countries with deforestation rate <0.22%/yr, we used the global mean rate in order to reflect that a REDD+ mechanism will likely provide an incentive to historically low-deforestation countries [42]. Given that deforestation in protected areas, IUCN I–II, has been reported to be substantially lower than of deforestation outside [43], we used 25% of deforestation rate for sites that are protected areas, or 75% of deforestation rate for unprotected sites. We focused on the net emissions assuming conversion to agriculture (which stores mean 8 t C/ha) [39].

#### Freshwater services

The estimated potential for provision of water to downstream populations was modeled on a global grid of 2,592 km^2^ hexagons based on spatially explicit maps of runoff [44], hydrological drainage direction [45], [46], and human population density [47]. Estimating hydrological services over large or unequal units (e.g., countries or watersheds) masks important variation within watersheds and conveys little useful information relevant to individual conservation sites. To capture this spatial variation, we modeled the flows of water from upstream source cells to human beneficiaries in downstream cells. Although the freshwater provided by habitats often acts as a supporting service for downstream ecosystems, we here focus on water most immediately available to people. Thus, a key step weights freshwater services according to the presence of human populations downstream. We began with global maps of runoff *f_i_* among cells *i* (available for use within *i* or in downstream cells); and demand *D_i_* (computed as total global water consumption [44] allocated among cells in proportion to human population of cell *i*), and applied the following equations:
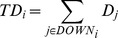
(1)

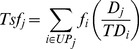
(2)

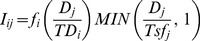
(3)

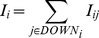
(4)Equation (1) computes the total demand *TD_i_* across all cells downstream of site *i*. Sets of cells upstream of *i* (*UP_i_*) or downstream (*DOWN_i_*) are computed from a global 30-arc-second drainage direction map [45], [46]. Equation (2) computes the total scaled flow *Tsf_j_* from all upstream cells into cell *j*. *Tsf* allocates flow from upstream cells to downstream cells *j* in proportion to demand in *j*, thus accounting for the fact that source cells generally supply water to more than one downstream cell. Equation (3) computes the water provision *I_ij_* of upstream cell *i* to downstream cell *j*. In so doing, it credits *i*'s contribution to *j* only to the point where *j*'s demand is met; no credit is given for contributions in excess of downstream demand. Finally, equation (4) sums up the total contribution *I_i_* of cell *i* to all downstream demand (See also [48]).

This is necessarily a coarse model but it captures much of the relevant spatial variation in elevation, precipitation, and nearby downstream population (which we expect to vary less within cells) and habitat (which varies within cells, but we account for that variation). We calculated the estimated water provision from a priority site as the mean water provision value among hexagon cells that the site covered (weighted by area of overlap).

For our second measure, water quality, we derived a water quality index based on the estimated influence of land cover on water quality (e.g., forests are more important for water quality than grassland) for any given site based on the potential water provision and land cover distributions at the site. Because the water quality implications of finely differentiated habitat types are poorly understood, we thus created a map of coarse land cover types from Global Land Cover 2000 (GLC2000) [40] combined with a global map of cloud forest [49], both having pixel size of 1 km^2^ or less (30 arc-second). We derived water quality coefficients for the broad land cover types, based on existing literature [21], [50]–[52]; e.g., forests effectively reduce surface erosion and increase water infiltration; wetlands effectively remove suspended solids, phosphorus, and nitrogen; and so on (See Table S1).

By mapping these coarse land cover types to GLC2000 land covers, each category in our merged land cover layer based on GLC2000 and cloud forest cover thus had quality coefficients (See Table S2). We only included natural land cover types in the analysis (i.e., excluded agriculture, bare area & artificial surface). We focused on the net water quality value assuming any conversion would be to agriculture. We computed a water quality index for each cell as the product of the water provision value for that cell times its area-weighted mean water quality coefficient. For example, a cell with water provision of 0.1 M m^3^/yr comprising half cloud forest and half sparse shrub cover would have a water quality index of 0.1[M m^3^/yr]×(0.5 [50% cloud forest]×(1.0 [cloud forest]−0.2 [agriculture])+0.5 [50% sparse shrub]×(0.35 [sparse shrub]−0.2 [agriculture]) = 0.02.

#### Option value

Here we used the number of narrow-ranged genera as a proxy for the evolutionary distinctiveness secured by a site's conservation and thereby its potential for retention of option value. This measure captures differences among biodiversity features over both geographic and phylogenetic space. We used two range thresholds in defining narrow-ranged genera: 1) range of less than 50,000 km^2^, commonly used as a threshold for endemism, e.g., for Endemic Bird Areas [53], and 2) range of less than 1,100 km^2^, the mean area of priority sites. Genus ranges were based on species distributions for all amphibians [15], [37], mammals [15], [38] and turtles [54]. There are 339 genera (encompassing 835 species) with ranges less than 50,000 km^2^, while 94 genera have ranges less than 1,100 km^2^ (135 species). The richness of narrow-ranged genera at sites was determined by spatial overlap of the distribution of genera with a 50 km buffer around the centroid of sites.

#### Cultural value

We used distribution maps of the world's languages [28], which, though imperfect, constitute the best available global data on language distribution [55]. We focused on both all languages and threatened languages, which are those language that are spoken by less 10,000 people [26]. The language richness of sites was determined by spatial overlap of the range maps that show the distribution of each language within a 50 km buffer around the centroid of sites.

### Comparison with null models (see also Table 1)

**Table 1:** Overview of analyses on comparison between performance of priority sites with the countries and ecoregions in which they are located.

	Global network of priority sites vs. random networks of sites in countries and ecoregions (Fig. 1)		Individual priority sites vs. mean of random networks in respective countries (Fig. 2)	
	Priority sites	‘Null model’	Priority sites	‘Null model’
CO_2_ emissions avoided	Overall value (t CO_2_/ha/year) for entire set of sites (n = 524)	Overall value (t CO_2_/ha/year) for random sets (n = 1,000) of 524 polygons (centroids with 18 km buffer) within countries holding priority sites	Site value (t CO_2_/ha) (per land area)	Overall mean±95%CL (t CO_2_/ha) for random polygons (centroids with 18 km buffer) in countries holding priority sites (per land area)
Freshwater services	Overall value per ha for entire set of sites	Overall value per ha in random sets (n = 1,000) of 524 polygons (centroids with 18 km buffer) within countries and ecoregions holding priority sites	Site value (water quality)	Overall mean±95%CL (Water quality) for random polygons (centroids with 50 km buffer) in countries holding priority sites (per land area)
Cultural value	Total number of languages for entire set of sites (centroids with 50 km buffer)	Number of languages in random sets (n = 1,000) of 524 polygons (centroids with 50 km buffer) within countries and ecoregions holding priority sites	Number of languages for each site (centroids with 50 km buffer) (all languages)	Mean number of languages±95%CL for random polygons (centroids with 50 km buffer) in countries holding priority sites (all languages)
Option value	Total number of narrow-ranged genera for entire set of sites (centroids with 50 km buffer)	Total number narrow-ranged genera in random sets (n = 1,000) of 524 polygons (centroids with 50 km buffer) within countries and ecoregions holding priority sites	Number of narrow- ranged genera for each site (centroids with 50 km buffer) (genera<50,000 km^2^)	No comparison made (see supplementary methods)

#### Performance of the entire network of priority sites (Fig. 1)

**Figure 1:**
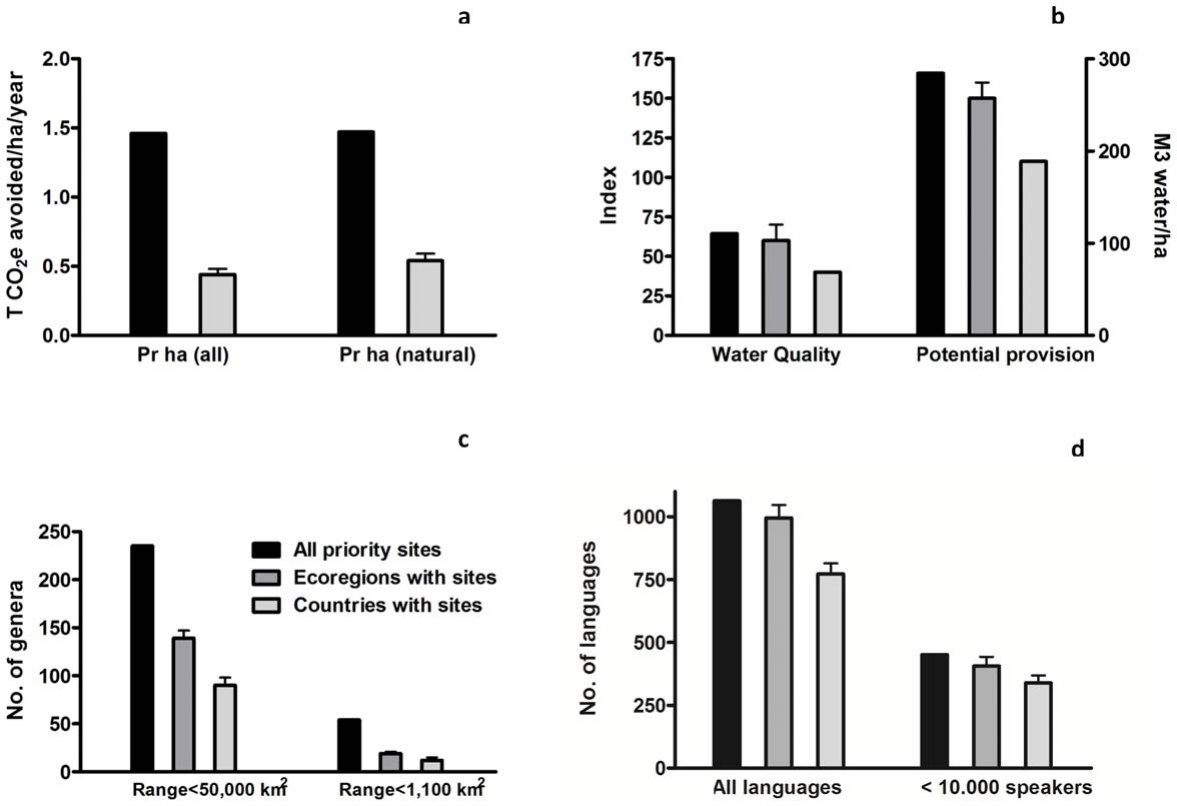
Ecosystem service delivery from priority sites compared to random. Estimated ecosystem service delivery from protection of the global network of priority sites (n = 524) compared to benefits expected at random from conservation of network of 524 sites within the same countries and ecoregions. a) Climate change mitigation through CO_2_ emissions avoided (estimated using national deforestation rates, thus only the country null model is used), b) Freshwater services (water quality and potential water provision), c) Option value measured as number of narrow-ranged genera (range less than 50,000 km^2^ and 1,100 km^2^), and d) Cultural value measured as the number of languages and threatened languages (less than 10,000 speakers). Columns denote 95% percentile and error bars denote 99% percentile of random networks of 524 sites in ecoregions and countries with priority sites (n = 10,000).

While the latitudinal gradient in species diversity is a recognized pattern, which partly explains why most critical sites for biodiversity are in the tropics, the global spatial patterns of ecosystem services are poorly understood. Our analytical approach reflects the lack of theoretical foundation for *a priori* expectations of spatial patterns for ecosystem services. Due to the use of global datasets there are uncertainties for values of specific sites and we therefore focus this study on the aggregate benefits for the entire network of priority sites. Taken together the summed values of the priority sites should be robust as there is no reason to expect systematic bias in values for the individual sites.

We compared estimated aggregate benefits from the network of critical conservation sites with benefits expected by chance from countries and ecoregions with priority sites, which gives an estimate of the relative global value of the benefits delivered by conserving priority sites. An alternative comparison using random distribution around the entire globe as a null model would have been excessively favorable to priority sites, while using the existing distribution of priority sites across countries (e.g., 63 in Mexico, 47 in Colombia) as a null model would be less relevant for assessing the global value of the network of priority sites.

For each ecosystem service we compared the overall estimated benefits for the entire set of 524 priority sites to the estimated benefits expected by chance from global networks of 524 sites within the countries and ecoregions [56] holding priority sites. For the two ecosystem services with values given as density (CO_2_ emissions avoided and freshwater services), we compared the overall mean for the polygons for the network of priority sites (i.e., total t CO_2_ emissions avoided/yr for the entire set of 524 priority sites) to global networks of 524 sites located randomly within the countries and ecoregions holding priority sites. Each site in the random network of sites constituted a polygon with 18 km radius to yield the same overall areas as for the network of priority sites. For CO_2_ emissions avoided we did not compare with ecoregions, because global deforestation data are only available at national levels. We repeated both analyses excluding the 51 priority sites which we had necessarily delineated using circles only, with qualitatively very similar results (Fig. S2).

The nature of the two other ecosystem services, cultural value and option value, are distinctively different as they constitute total counts – rather than densities – of overlap with ranges of either narrow-ranged genera or languages. Thus, for both the priority sites and null models, we used polygons of 50 km radius (buffer around centroid of sites) to estimate potential richness of narrow-ranged genera and languages, respectively. Consequently, we sampled sets (n = 10,000 sets) of 524 randomly distributed sites within the countries and ecoregions holding priority sites. We compared these with the performance of circles of radius 50 km around the 524 actual priority site centroids.

#### Performance of individual priority sites (Fig. 2)

**Figure 2:**
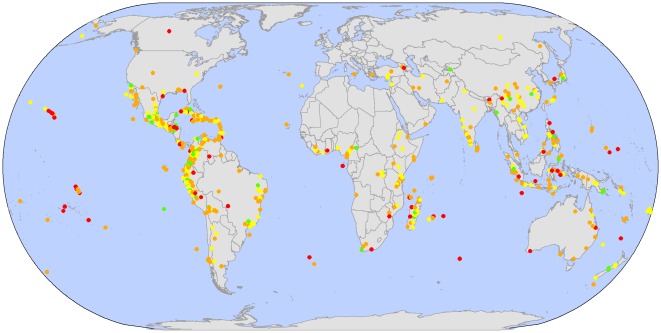
Location of the global network of priority sites and their relative performance compared to country mean. Comparison of the delivery of the ecosystem services for each of the priority sites to the mean±95% confidence interval within the same countries. The ecosystem services included are a) climate mitigation through CO_2_ emissions avoided, b) water quality, and c) cultural value as number of languages. Green sites perform better than national means for all three services (9%), yellow for two (35%), orange for one (45%), and red for none (11%). (Option value is excluded as mean±95% confidence interval could not be derived, see supporting methods).

Given uncertainties with the values from specific sites due to resolution of the global datasets, we avoid going into much detail or recommendations based on specific sites. However, to reveal overall spatial global pattern of higher-performing priority sites, we mapped each priority site in terms of the number of ecosystem services for which the site performed better than the mean±95% confidence interval of the total random sites within each country in which it was located (Fig. 2).

We used countries as the units for these comparisons rather than ecoregions because countries are far more often the units in which decisions are made. In addition, values were available for all four services for countries (including CO_2_ emissions avoided, a measure not available for ecoregions). For this comparison, we restricted the analysis to the most relevant aspect of each of the four ecosystem services:

CO_2_ emissions (per land area rather than per forest area to assess relative value of potential CO_2_ emissions avoided for priority sites).
Freshwater service (water quality rather than water provision as intact ecosystems are particularly important for water quality).
Cultural value (all languages are more broadly indicative of cultural value than the subset of threatened languages).
Option value (genera with ranges >50,000 km^2^ encompass more unique evolutionary history than the subset of genera with ranges >1,100 km^2^). Despite using this more inclusive definition of option value, this analysis yielded many zero values (57% of priority sites and 85% of random sites), reflecting the fact that many sites cover no narrow-ranged genera, which precluded calculation of reliable confidence limits around the mean. Thus, comparison was not made for option value.

### Economic benefits versus costs

#### Benefits from CO_2_ emissions avoided

A recent review of forest carbon market prices and the voluntary carbon market suggests a range from US$ 1–15/t CO_2_e for forest carbon with a mid-range estimate of US$ 7.50 [43]. We applied estimates of US$ 5 and US$ 10/t CO_2_e, which is multiplied by annual CO_2_ emissions avoided (t CO_2_/yr) to get the overall potential yield. For this analysis we used carbon stock in forest by restricting the global carbon map to forest by using a global land cover layer [40]. In comparison, estimates on the social cost of carbon, or the marginal damage costs associated with CO_2_ emissions, has been surveyed with median values of US$ 66 to US$130 per t C (US$ 18 to US$ 35/t CO_2_e) in 2010 US dollars (depending on discounting assumptions) [57].

#### Costs of creating and managing the network of priority sites as protected areas

The costs of a network of priority sites are a combination of acquisition costs and ongoing costs of management. Acquisition cost can vary widely depending on whether protected areas are established on public or private lands [31]; while opportunity costs will be borne in both cases, often only the latter case will involve payments by governments. Given this variation, we estimate the total costs as a range from management cost alone (no acquisition costs) to management costs plus acquisition costs (full acquisition costs) [31].

#### Management costs

We used a model [31] that predicts the cost of effective management for terrestrial areas as protected areas by using information on priority sites (site area, multiple use of protected area, and percentage of site within 10 km of road) and the countries they are found in (GDP [58], Human Development Index [59]).

#### Acquisition costs

As a proxy for acquisition costs we use opportunity costs, based on a global map of economic rents from agricultural lands at 5′ resolution [32]. We used the ‘potential agricultural rents’ layer, which is not restricted to the area actually occupied by each crop and therefore includes, e.g., large wilderness areas, and we converted income to returns by using a profit margin of 15% [60]. The US$ 2000 values were converted to US$ 2008 values as management costs were estimated in 2008 (GDP). For the comparison of benefits versus creation and management costs for the set of priority sites, we used the 319 priority sites in developing countries for which data are available for all three measures.

## Results

Overall, the aggregated values for the network of priority sites performed significantly better for all four ecosystem services than the random networks of sites in the countries and ecoregions where the sites are found (Fig. 1). The protection of priority conservation sites would prevent emissions of 1.5 t CO_2_e/ha/yr, which is significantly higher than random (P<0.01), and approximately three times the emissions reduction expected by chance from networks of sites in the same countries (Fig. 1a). Since we only had deforestation rates for countries, we could not make this comparison for ecoregions. However, carbon storage is significantly higher (P<0.01) for the priority sites than for the random networks for both countries and ecoregions (Fig. S1). In aggregate the priority sites hold 83.3 t C/ha of natural area, while the means for the random networks are 38.6 t C/ha and 56.0 t C/ha for countries and ecoregions, respectively.

Protection of priority sites would deliver substantially greater freshwater services than other sites within the same countries or ecoregions (Fig. 1b). The net contribution to water quality from conservation of these sites is higher than expected at random (P<0.05); 1.3 times higher than for countries and 1.8 times higher than for ecoregions. Similarly, the estimated water provision from conservation of these sites is significantly higher than expected (P<0.01); 1.2 and 1.8 times higher than for countries and ecoregions, respectively. These findings for both carbon storage and freshwater services are overall similar if the comparison is only made with the subset of 473 priority sites with well defined boundaries (i.e., excluding the 51 sites with circular buffers. See Fig. S2).

Despite the fact that priority site identification was conducted wholly at the species level, safeguarding these priority sites would also protect significantly more narrow-ranged genera (P<0.01; Fig. 1c). The priority sites cover the range of 54 and 235 genera with ranges less than 1,000 km^2^ and 50,000 km^2^, respectively, compared to a mean of 12.8 and 121.4 for countries and 6.7 and 71.8 for ecoregions, respectively.

Finally, we found priority sites to lie in areas of significantly higher linguistic diversity for both all languages and threatened languages (P<0.01; Fig. 1d). The priority sites cover the range of 1,063 languages and 451 threatened languages compared to a mean of 672 and 271 for countries and 880 and 323 for ecoregions, respectively.

The aggregate values for the entire network of priority sites clearly encompass considerable variation in delivery of ecosystem services among individual sites. Figure 2 shows how priority sites perform compared to random sites for some ecosystem services in their country. Provision of ecosystem services among individual sites varies considerably (Fig. 2). Some priority sites are extremely important for several ecosystem services, while other sites do not outperform random sites for some ecosystem services in the country they are found in. Figure 2 reveals that the priority sites than perform relatively well are those located in tropical mountains such as the Mexican Sierras, tropical Andes, Afromontane systems, and Indian Western Ghats. The geographical pattern of priority sites for each ecosystem service considered individually shows the same general overall pattern (See also Fig. S3 and Table S3).

For one ecosystem service – carbon storage – we can predict potential financial benefits for comparison to the financial costs of protecting these sites. We compared the 319 priority sites in developing countries that have data for benefits, management costs and opportunity costs. Conservation of these 319 priority sites in developing countries could yield an estimated revenue of US$ 165–331 million annually under a carbon market mechanism to mitigate climate change, assuming a carbon price of US$ 5–10/t CO_2_e/yr [18]. This is necessarily a simplified measure given the underlying data and transaction costs were not considered. By comparison, our estimate of the cost of creating and managing these 319 priority sites as a protected area network range from US$ 304 million per year (management costs for the network) to US $2,411 million per year (management costs+full acquisition costs) suggesting that REDD+ revenue alone might be sufficient to finance their ongoing conservation (if there are no acquisition costs). However, foregone agricultural opportunities from safeguarding these sites' costs could be much larger, at US$ 2,411 million, 7–15 times higher than the potential REDD+ revenue for these 319 sites within developing countries.

## Discussion

Overall, we found that the network of priority sites performed significantly better than expected for all four ecosystem services. We found that the potential for avoidance of CO_2_ emissions was disproportionately higher for the priority sites. Several factors drive this result. The overall carbon stock per unit area in priority sites is 2.2 times higher than median in countries holding priority sites (1.5 times higher than ecoregions holding priority sites; Fig. S1) mainly because priority sites have a higher proportion of forest cover (63%) than their encompassing countries and ecoregions (36/44%, respectively; all subsequent comparisons follow this same format) and more carbon-dense forest (1.4/1.2 times more C per forest area). The threatened species that trigger priority site identification also tend to be in areas with greater habitat loss rates: 59% of the sites lie within high-deforestation countries [42].

The disproportionate delivery of freshwater can be attributed to four factors. First, priority sites hold a relatively high proportion of forest, a land cover of particular importance for water quality (76% and 44% higher for country and ecoregion comparisons, respectively). In addition, priority sites overall were situated in areas with more people (mean 134 people/km^2^ within 50 km of priority sites vs. 52/71 people/km^2^), with higher elevations [61] (mean elevation of 1,050 m for priority sites vs. 648/760 m) and thus more downstream area, and with higher precipitation [62] (mean precipitation at priority sites of 1,461 mm/year vs. 726/958 mm/year).

The priority sites, which are identified solely based on species-level information for one or more Endangered or Critically Endangered species, would also protect a disproportionate share of narrow-ranged genera. This finding suggests that protecting these priority sites would provide a higher potential for preserving unique evolutionary history for humanity's future use. Clearly it is difficult to provide a robust proxy for ‘option value’ – the potential value to society – as these values are not yet realized. Nevertheless, a compelling argument can be made that maximizing the retention of phylogenetic diversity (PD) should also maximize option value, as well as diversification and adaptation of the species in a future of climatic change [23]. It should be noted that we used only one measure of option value. Alternative measures of other aspects of option value, e.g. [23], including biodiversity measures related to specific current uses such as agricultural biodiversity or specific groups of organisms with strong records of pharmaceutical compounds might have revealed different findings – although the lack of data renders such measures impossible to apply at the global level at present.

We found priority sites to lie in areas of significantly higher linguistic diversity of both all languages and threatened languages (i.e. those spoken by <10,000 people). Linguistic diversity is positively correlated with forest area and maximum altitude in countries [29], which might contribute to the observed pattern. While the link between safeguarding priority sites and cultural value through preserving languages is complex, these findings do suggest a potential importance of priority site conservation for the maintenance of cultural value. While this does not imply that protecting these sites necessarily would help conserve their threatened human cultures, an inclusive approach to conservation action at these sites that are disproportionally important for local human cultures could help maintain cultural value. These findings also emphasize the importance of collaboration with indigenous people in planning and implementing conservation efforts. It should be noted that we have focused on one particular measure of cultural value for which data were available, and an analysis using another measure of cultural value in the broader sense (including, e.g. recreational value) might reveal other findings.

Provision of ecosystem services among individual sites varies considerably (Fig. 2, see also Fig. S3 and Table S3). While the aggregated values of the priority sites should be robust (as there is no reason to expect systematic bias in values for the individual sites), there are considerable uncertainties with the values from specific sites due to resolution of the global datasets. Consequently, we caution against inferring detail for specific sites, instead focusing on the overall spatial pattern of priority sites compared to random sites within countries. Those sites which provide most disproportionate ecosystem service benefits compared to alternative sites within their respective countries tend to be in tropical mountains. Such regions are characterized by tropical forest remnants, rapid deforestation, high rainfall, and large human populations, which combine to drive this result. Those sites which provide fewest ecosystem service benefits lie mainly in small oceanic islands (e.g., in the Gulf of California, the Lesser Antilles, and the Indian Ocean islands), which, conversely, often hold xeric habitats, low rainfall, and sparse human population. Further analysis at a finer scale with local/regional data will be needed to assess the value of specific priority sites for delivery of ecosystem services.

This analysis estimated that the conservation of these priority sites will provide disproportionate delivery of ecosystem service benefits in the non-monetary sense. An important question is how the financial benefits of these ecosystem services will compare to the financial cost of protecting them. Unfortunately, data on economic benefits for most ecosystem services are unavailable in most regions. Thus, we can only predict potential financial benefits of one ecosystem service – carbon storage – for comparison to the financial costs of protecting these sites. Nevertheless, the estimate is useful in illustrating the magnitude of the potential financial benefits arising from carbon storage. Our lower range estimate of the cost of protecting the global network of priority sites predicts management costs for the network are on the same order of magnitude as this potential economic benefit, suggesting that REDD+ revenue alone could be sufficient to finance their ongoing conservation. However, the upper range measure, which includes acquisition costs in terms of foregone agricultural opportunity from safeguarding these sites, suggests that costs would be much larger (7–15 times) than the total potential REDD+ revenue for these sites. Although this result is consistent with other analyses of REDD+ benefits relative to conservation costs [63], it probably overestimates the value of opportunity costs given maximum productivity is assumed, which often will not be the case in most regions. On the other hand, the measure of agricultural rents does not capture other aspects of opportunity costs such as lost opportunities for hydroelectric development, road building, mining etc., which in some places may be of considerable value [64].

That potential REDD+ revenue from safeguarding all priority sites would be exceeded by opportunity costs is unsurprising, given that carbon storage is associated with high-carbon ecosystems such as forests and thus non-forest sites will tend to perform poorly. Thus, although REDD+ funding constitutes a source of finance that can provide incidental benefits to conservation, a narrow focus on the carbon value of conservation areas could potentially neglect many valuable conservation sites and their associated ecosystem services. A more comprehensive economic analysis – albeit one for which global data are not yet available – would also include the estimated economic benefits from various other ecosystem services arising from the intact habitats in these priority sites such as clean freshwater, climate change adaptation, ecotourism, and others. When the full range of benefits is taken into account, the economic benefits of conservation often exceed costs at both global and national scales [9], [65], [66]. Moreover, there are numerous other reasons to protect these sites, not least the option and cultural values estimated here, in addition to that of preventing the extinction of the species themselves.

While values for individual sites are uncertain in a global analysis, the aggregated values are robust. Here our results are surprisingly consistent across four disparate ecosystem services and varied data sources. These critical conservation sites, essential for halting imminent species extinctions, may be also effective choices for delivery of ecosystem services for human well-being.

## Supporting Information

Figure S1 **Estimated carbon storage in natural land covers from protection of the global network of priority sites (n = 524) compared to null models of predicted benefits from conservation within the same countries and ecoregions.** Columns denote 95% percentile and error bars denote 99% percentile of random networks of sites in ecoregions and countries with priority sites (n = 10,000).
(TIF)

Figure S2 **Ecosystem service delivery from protection of global network of priority sites (n = 473, i.e., excluding those 51 priority sites where boundaries could not be defined based on existing polygons) compared to null model within the same countries and ecoregions: a) estimated carbon storage and, b) estimated freshwater services.** Columns denote 95% percentile and error bars denote 99% percentile of random networks of sites in ecoregions and countries with priority sites (n = 10,000).
(TIF)

Figure S3 **The relative ecosystem service delivery of priority sites compared to random sites in the country in which they are located. a) CO2 emissions avoided (per land area).** b) Water quality to downstream populations. c) Cultural value measured as number of languages in and near sites. Priority sites that are significantly better (green), worse (red), and equal to (white) than mean ±95% confidence interval of random sites. Data deficient sites are black.
(TIF)

Table S1 **Water quality coefficients for main land cover types.**
(DOC)

Table S2 **Water quality coefficients for the broad land cover types.**
(DOC)

Table S3 **Provision of ecosystem services from individual priority sites compared to mean of country in which they are located.**
(DOC)
