# Supplementary figures and images for: Conserving Critical Sites for Biodiversity Provides Disproportionate Benefits to People
Source: PLoS One. 2012 May 30;7(5):e36971. doi: 10.1371/journal.pone.0036971 (PMC3364245; doi:10.1371/journal.pone.0036971)

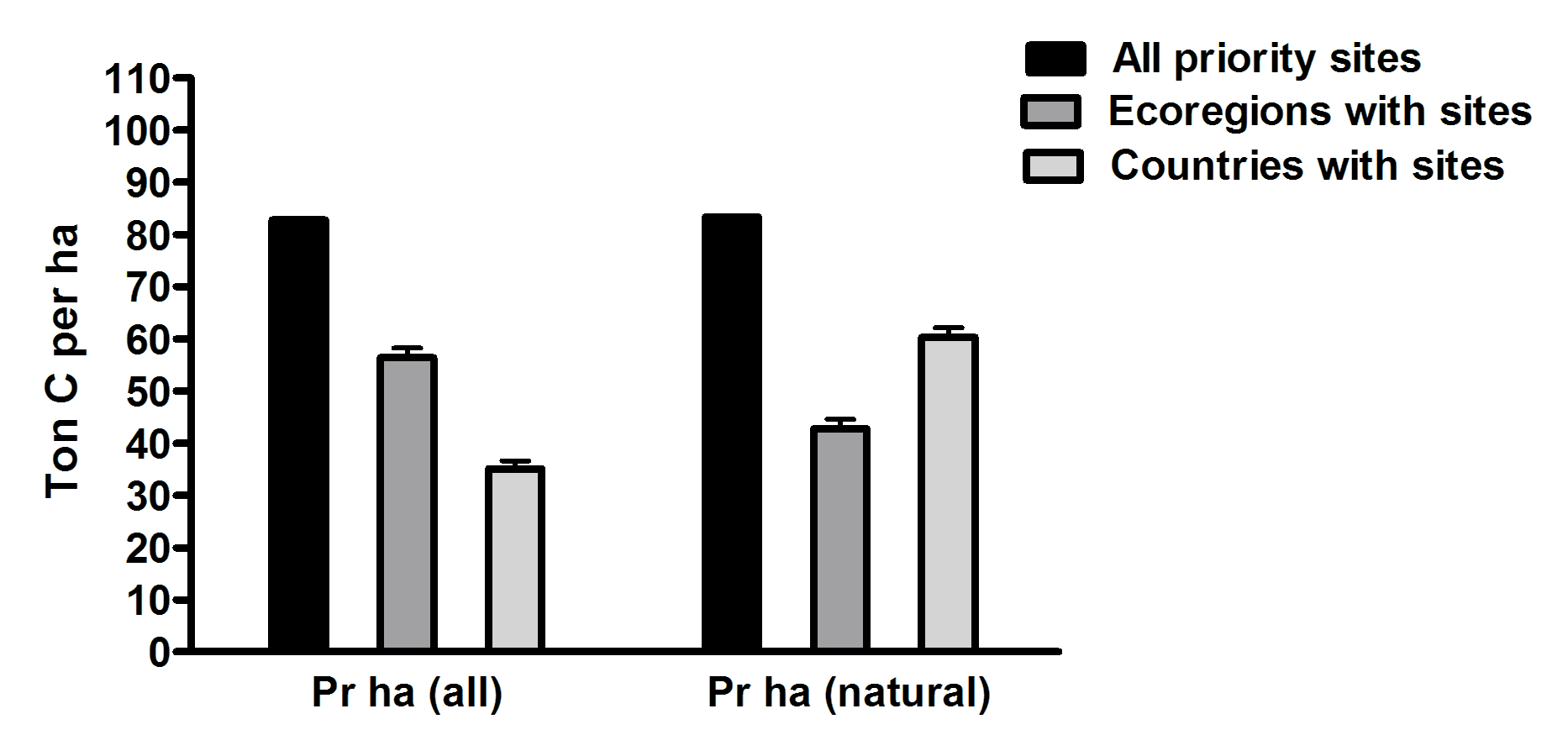

Supplement: Figure S1 — Estimated carbon storage in natural land covers from protection of the global network of priority sites (n = 524) compared to null models of predicted benefits from conservation within the same countries and ecoregions. Columns denote 95% percentile and error bars denote 99% percentile of random networks of sites in ecoregions and countries with priority sites (n = 10,000). (TIF) [file pone.0036971.s001.tif]

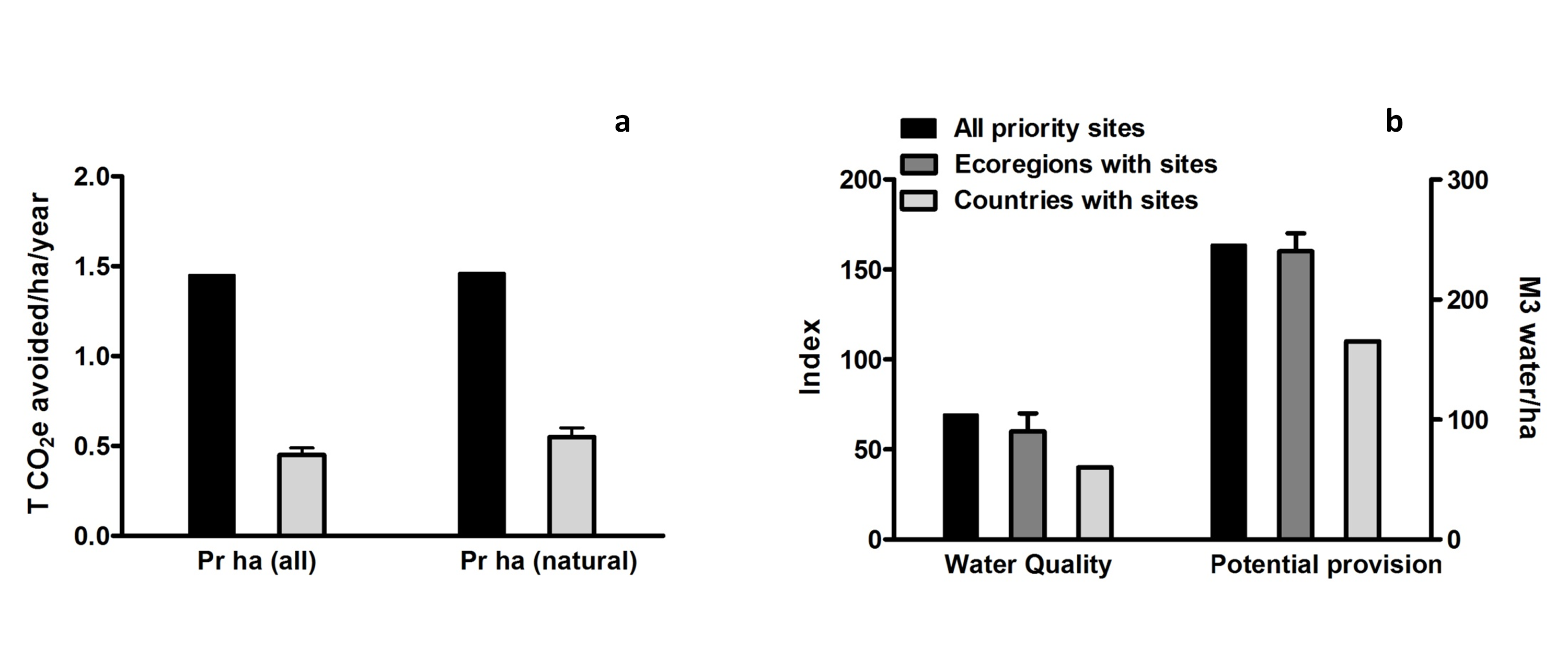

Supplement: Figure S2 — Ecosystem service delivery from protection of global network of priority sites (n = 473, i.e., excluding those 51 priority sites where boundaries could not be defined based on existing polygons) compared to null model within the same countries and ecoregions: a) estimated carbon storage and, b) estimated freshwater services. Columns denote 95% percentile and error bars denote 99% percentile of random networks of sites in ecoregions and countries with priority sites (n = 10,000). (TIF) [file pone.0036971.s002.tif]

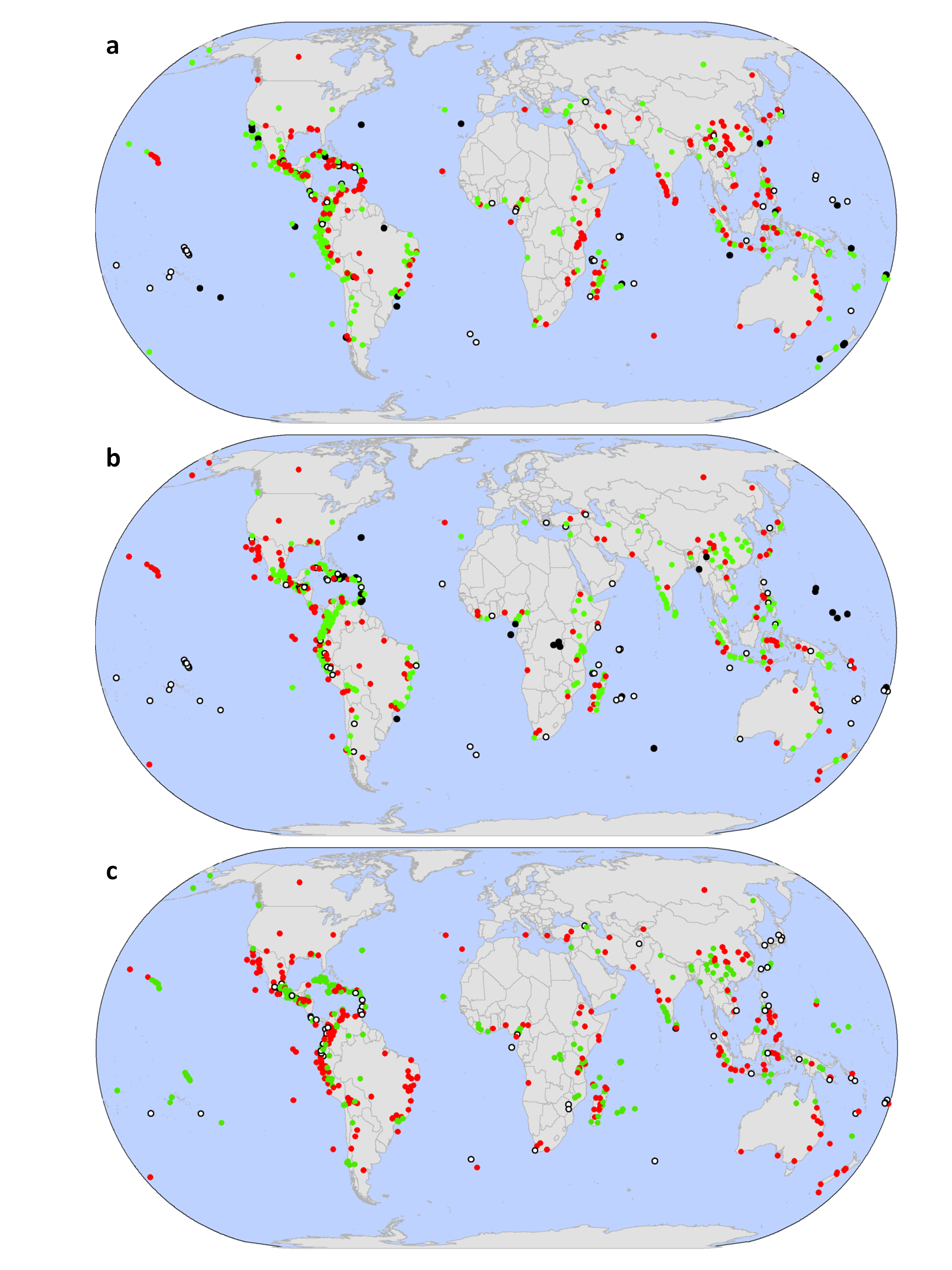

Supplement: Figure S3 — The relative ecosystem service delivery of priority sites compared to random sites in the country in which they are located. a) CO2 emissions avoided (per land area). b) Water quality to downstream populations. c) Cultural value measured as number of languages in and near sites. Priority sites that are significantly better (green), worse (red), and equal to (white) than mean ±95% confidence interval of random sites. Data deficient sites are black. (TIF) [file pone.0036971.s003.tif]
