# Supplementary material for: Conserving Critical Sites for Biodiversity Provides Disproportionate Benefits to People
Source: PLoS One. 2012 May 30;7(5):e36971. doi: 10.1371/journal.pone.0036971 (PMC3364245; doi:10.1371/journal.pone.0036971)
Supplement: Table S1 — Water quality coefficients for main land cover types. (DOC) [file pone.0036971.s004.doc]

**Table S1 Water quality coefficients for main land cover types**

| **Broad Land cover type** | **Water quality** |
| --- | --- |
| Wetlands | 1.0 |
| Forest | 1.0 |
| Cloud forest | 1.0 |
| Water | 0.8 |
| Grasslands and Shrublands | 0.6 |
| Snow and ice | 0.3 |
| Agriculture | 0.2 |
| Bare areas & artificial surfaces | 0.1 |
