# Supplementary material for: Conserving Critical Sites for Biodiversity Provides Disproportionate Benefits to People
Source: PLoS One. 2012 May 30;7(5):e36971. doi: 10.1371/journal.pone.0036971 (PMC3364245; doi:10.1371/journal.pone.0036971)
Supplement: Table S2 — Water quality coefficients for the broad land cover types. (DOC) [file pone.0036971.s005.doc]

**Table S2 Water quality coefficients for the broad land cover types**

| **Code** | **Category** | **Broad Land cover type** | **Coefficient** |
| --- | --- | --- | --- |
| 1 | Tree Cover, broadleaved, evergreen | Forest | 1.00 |
| 2 | Tree Cover, broadleaved, deciduous, closed | Forest | 1.00 |
| 3 | Tree Cover, broadleaved, deciduous, open | Forest | 1.00 |
| 4 | Tree Cover, needle-leaved, evergreen | Forest | 1.00 |
| 5 | Tree Cover, needle-leaved, deciduous | Forest | 1.00 |
| 6 | Tree Cover, mixed leaf type | Forest | 1.00 |
| 7 | Tree Cover, regularly flooded, fresh water | Wetlands | 1.00 |
| 8 | Tree Cover, regularly flooded, saline water | Wetlands | 1.00 |
| 9 | Mosaic: Tree Cover / Other natural vegetation | 50% Forest; 50% Grasslands and Shrublands | 0.80 |
| 10 | Tree Cover, burnt | 50% Forest; 50% Bare areas & artificial surfaces | 0.55 |
| 11 | Shrub Cover, closed-open, evergreen | Grasslands and Shrublands | 0.60 |
| 12 | Shrub Cover, closed-open, deciduous | Grasslands and Shrublands | 0.60 |
| 13 | Herbaceous Cover, closed-open | Grasslands and Shrublands | 0.60 |
| 14 | Sparse herbaceous or sparse shrub cover | 50% Grasslands and Shrublands; 50% Bare areas & artificial surfaces | 0.35 |
| 15 | Regularly flooded shrub and/or herbaceous cover | Wetlands | 1.00 |
| *16* | *Cultivated and managed areas* | *Agriculture* | *0.20* |
| *17* | *Mosaic: Cropland / Tree Cover / Other natural vegetation* | *Agriculture* | *0.20* |
| *18* | *Mosaic: Cropland / Shrub and/or grass cover* | *Agriculture* | *0.20* |
| 19 | Bare Areas | Bare areas & artificial surfaces | 0.10 |
| 20 | Water Bodies | Water | 0.80 |
| 21 | Snow and Ice | Snow and ice | 0.30 |
| *22* | *Artificial surfaces and associated areas* | *Bare areas & artificial surfaces* | *0.10* |
| 23 | No Data |  | 0.00 |
| 24 | Cloud forest | Cloud forest | 1.00 |
