# Supplementary material for: Conserving Critical Sites for Biodiversity Provides Disproportionate Benefits to People
Source: PLoS One. 2012 May 30;7(5):e36971. doi: 10.1371/journal.pone.0036971 (PMC3364245; doi:10.1371/journal.pone.0036971)
Supplement: Table S3 — Provision of ecosystem services from individual priority sites compared to mean of country in which they are located. (DOC) [file pone.0036971.s006.doc]

**Table S3 Provision of ecosystem services from individual priority sites compared to mean of country in which they are located**

|  | **Priority sites versus country mean** | | | |
| --- | --- | --- | --- | --- |
| **Ecosystem service** | Site better | Equal | Site worse | No data |
| CO_2_ emissions | 40% (209) | 10% (51) | 46% (239) | 5% (25) |
| Water quality | 50% (262) | 15% (79) | 30% (159) | 5% (24) |
| Cultural value (all languages) | 40% (211) | 12% (61) | 48% (252) | 0% (0) |
